# Influence of Intragastric Administration of Traditional Japanese Medicine, Ninjin'Yoeito, on Cerebral Blood Flow via Muscarinic Acetylcholine Receptors

**DOI:** 10.1155/2021/9930023

**Published:** 2021-08-07

**Authors:** Nobuhiro Watanabe, Kaori Iimura, Harumi Hotta

**Affiliations:** Department of Autonomic Neuroscience, Tokyo Metropolitan Institute of Gerontology, Tokyo, Japan

## Abstract

Ninjin'yoeito (NYT) is a traditional medicine that has been used for mitigating physical frailty, such as fatigue and anorexia, as well as for cognitive dysfunction. Maintenance of adequate cerebral blood flow (CBF) is important for preventing cognitive dysfunction. The present study aimed to examine the effect of NYT on CBF. Male C57BL/6 J mice were anesthetized with urethane and were artificially ventilated. We measured CBF in the neocortex with laser-speckle contrast imaging for 10 min before administration and 60 min after administration. We administered NYT solution (0.25, 0.5, 1, and 2 g/kg) or vehicle (distilled water; DW) over 5 min via an intragastric catheter. We surgically transected the vagus nerve to investigate its contribution as a neural pathway and intraperitoneally injected atropine to block muscarinic acetylcholine receptors. Finally, we tested the CBF response to cutaneous brushing stimulation applied to the left hind paw (30 sec). CBF decreased after DW administration, starting from 30 min onward, whereas CBF did not change after NYT. The averaged CBF change following DW administration differed from that following NYT (1 g/kg) but not from those following the other doses of NYT. Arterial pressure was not affected by either solution. CBF after NYT (1 g/kg) was not affected by vagotomy but was lower following additional atropine. In response to brushing stimulation, CBF in the right (contralateral) parietal cortex increased. The magnitude of CBF increase following NYT was greater than that following DW. These results suggest that NYT prevents CBF decrease via cholinergic activation independent of vagal activity and enhances the CBF response to somatosensory stimulation.

## 1. Introduction

Ninjin'yoeito (NYT) is a traditional Japanese medicine, consisting of 12 crude drugs [1]. Clinically, NYT is used for mitigating physical frailty, such as fatigue, cold limbs, anorexia, and anemia. Such effects are thought to affect neural, endocrine, and immune cells [1]. Additionally, a recent report has shown that NYT improved cognitive decline in Alzheimer's disease (AD) [2]. NYT has also been shown to be effective for improving cognitive impairment in experimental animals [1, 3]. As to its mechanism, NYT's effects on the cerebral cholinergic system have been suggested [1, 4].

The cerebral cholinergic system is important not only for cognitive function [5] but also for cerebral vasodilation [6–11]. In patients with dementia, the resting cerebral blood flow (CBF) is lower [12–16], and the increase in CBF by sensory stimulation is attenuated [17, 18] compared with healthy controls. However, cholinesterase inhibitors increase CBF, improving cognitive decline [19, 20]. Thus, we expected that NYT would increase CBF if NYT acts on the cerebral cholinergic system. To our knowledge, the effect of NYT on CBF is yet to be studied.

We then hypothesized that NYT activates the cerebral cholinergic system and increases CBF. To test this hypothesis, we first examined the effect of intragastric administration of NYT on resting CBF in anesthetized mice. This experiment revealed a difference from vehicle; thus, we further investigated the possibility that neural information from the gastrointestinal tract and muscarinic acetylcholine (mACh) receptors contribute to the mechanism. Finally, the effect of NYT administration on somatosensory stimulation-induced CBF increase was examined.

## 2. Materials and Methods

### 2.1. Animals

The present study consisted of four experiments. In total, 32 male C57BL/6 J mice (7–9 months old and 28.8–41.3 g) were used. The number of animals used in each experiment is summarized in Table 1. Animals in the present study were purchased at 4 weeks of age (Japan Charles River, Yokohama, Japan) and were kept at the Tokyo Metropolitan Institute of Gerontology until the experiment. They were housed in individually ventilated cages and had access to a commercial pelleted diet (CRF-1, Oriental Yeast Co., Ltd., Tokyo, Japan) and filtered tap water with 2 ppm of chlorine *ad libitum*. The environment in the vivarium was maintained at 22 ± 1°C and 55 ± 5% of relative humidity with a 12-hour light cycle (lights off at 20 : 00 h). The protocol of the present study was approved by the animal care and use committee of the Tokyo Metropolitan Institute of Gerontology (animal ethics committee: approval number 18034–2) and in accordance with the “Guidelines for Proper Implementation of Animal Experiments” established by the Japan Society for the Promotion of Science in 2006. All experimental procedures were carried out only after the protocol was approved.

We first anesthetized the animals with sevoflurane (4% for 3 min), then administered a urethane injection (1.4 g/kg). We judged the sufficient depth of anesthesia by the loss of corneal and withdrawal reflexes. When necessary, we additionally administered 4%–19% of the initial dose of urethane, or the animals inhaled sevoflurane (0.2%–0.8%) during surgery. We cannulated the trachea and artificially ventilated the mice (MiniVent Type 845, Harvard Apparatus, MA, USA). In part of the cohort, we continuously recorded arterial blood pressure via a catheter implanted in the right femoral artery (Table 1) and calculated mean arterial pressure. To maintain systemic conditions, core body (rectal) temperature was maintained at 37.0–37.5°C (ATB-1100, Nihon Kohden, Tokyo, Japan), and we subcutaneously administered saline via an implanted needle (30 gage) at a rate of 3 *μ*L/min with an infusion pump (ESP-64, EICOM, Kyoto, Japan). We began data collection at least 20 min after the surgery. Mice were euthanized by injecting an overdose of pentobarbital at the end of each experiment.

### 2.2. Ninjin'yoeito

The NYT was supplied by Tsumura & Co. The formula to produce 6 g of NYT extract powder was composed of a mixture of 12 dried natural components: Rehmanniae radix (4 g), Angelicae acutilobae radix (4 g), Atractylodis rhizoma (4 g), Poria (4 g), Ginseng radix (3 g), Cinnamomi cortex (2.5 g), Polygalae radix (2 g), Paeoniae radix (2 g), Citri unshiu pericarpium (2 g), Astragali radix (1.5 g), Glycyrrhizae radix (1 g), and Schisandrae fructus (1 g). The mixture was boiled for 1 h, after which the resultant extract was filtered, concentrated, and spray-dried to produce the NYT extract powder (lot number: 362113100). The NYT powder was suspended in distilled water (DW; Otsuka Pharmaceutical Co., Ltd., Tokyo, Japan), and the NYT solution (0.25, 0.5, 1, and 2 g in 5 mL of DW) was prepared and intragastrically administered (5 mL/kg of body weight). DW was used as control (0 g).

### 2.3. Intragastric Administration

Polyethylene tubing (SP-10, Natsume Seisakusho, Tokyo, Japan) was implanted for solution administration in the lumen of the stomach via the mouth. The solution was placed in a gastight syringe (#1002, Hamilton Co., Reno, NV, USA) and administered over 5 min with an infusion pump (ESP-64, EICOM). A maximum of three doses was administered per mouse, and the order of administration was randomized. The interval between administrations was at least 60 min.

### 2.4. Cerebral Blood Flow Measurement

The head of each mouse was fixed to a stereotaxic instrument with ear bars (SR-5M-S, Narishige, Tokyo, Japan). The scalp was excised, and the skull was exposed. The skull was kept intact, and liquid paraffin oil was applied to prevent drying. CBF was measured using a laser-speckle contrast imager consisting of an infrared semiconductor laser (wavelength at 785 nm) and a charge-coupled device camera (moor LFPI2; Moor Instruments, Devon, UK). The device was set above the dorsal head of the mouse, and the zoom was adjusted to include the whole brain. The field of view was approximately 261.8 mm^2^ (18.7 mm × 14.0 mm). One plane image consisted of 752 × 580 pixels, and the pixel size was approximately 25 *μ*m. Images were acquired at a rate of 1 frame per second or 10 sec with 20 ms of exposure time.

To quantify the regional CBF, blood flow data were extracted by a region of interest (ROI) with a diameter of 1.5 mm (moorFLPI Review V5.0, Moor Instruments). The ROI was on the right parietal cortex (AP = 0 to −1.5 mm from the Bregma and *L* = 0.5 to 2 mm from the midline) [21].

### 2.5. Influence of NYT Administration on Resting CBF (Experiment 1)

CBF was measured for 10 min before intragastric administration of solution (preadministration baseline). After that, the solution administration was started, and CBF measurement was continued for 60 min. CBF was acquired as an image at an average of every 10 sec. The acquired images were averaged every 10 min. CBF change following solution administration was calculated as the rate of change (%) with respect to the preadministration baseline. Similarly, mean arterial pressure was averaged every 10 min, and the change rate (%) following solution administration was calculated.

### 2.6. Nerve Transection (Experiment 2)

To examine the possibility that resting CBF following intragastric administration of NYT is affected by sensory information from the gastrointestinal tract via the vagus nerve, the bilateral vagus nerve was surgically transected at the cervical level (*n* = 7). Considering the possibility that information about administered NYT ascends the spinal cord, spinalization was performed at the T2/3 level in addition to the vagotomy (*n* = 2). We commenced CBF recording more than 50 min after nerve transection. In experiment(s) 2 (and 3), the CBF was recorded and analyzed as performed in experiment 1.

### 2.7. Acetylcholine Receptor Blocker (Experiment 3)

To examine the contribution of cerebral acetylcholine receptors to the influence of NYT administration on resting CBF, a blood–brain barrier-permeable dose of a mACh receptor antagonist atropine (ATR; atropine sulfate salt monohydrate, Sigma-Aldrich, Inc., St. Louis, MO, USA) was used. We intraperitoneally injected ATR (5 mg/kg that does not affect arterial blood pressure [22]) and commenced CBF measurement 30 min after injection.

### 2.8. Effect of NYT on CBF Increase Induced by Brushing Stimulation (Experiment 4)

For somatosensory stimulation, brushing stimulation was applied to the mouse's left hind paw. The stimulation was manually applied from the proximal to distal paw with a paintbrush at the frequency of 2 strokes/sec for a period of 30 sec and was paced using a metronome rhythm. The stimulation was performed at 10–16 min following the commencement of DW or NYT (1 g/kg) administration. For the setting of experiment 4, the route from a solution-filled syringe to the mouth was masked; thus, the experimenter (K. I.) who performed the brushing stimulation was blinded to the type of solution administered to the mouse.

CBF images were acquired every 1 sec in Experiment 4. The acquired images were averaged every 5 sec, and CBF data were extracted by ROI. Maximum CBF change during stimulation (%) was calculated with respect to CBF value immediately before the onset of brushing stimulation (100%).

### 2.9. Statistical Analysis

To be conservative, we used nonparametric tests for the statistical analyses [23], given the sample size in the present study was relatively small (6 or 7 mice/group). This sample size was selected based on the previous studies that examined the effect of cerebral cholinergic system activation on CBF in anesthetized rodents [10, 24]. The analysis was performed using statistics software (Prism6, GraphPad Software Inc., La Jolla, CA, USA). The time course of the resting CBF change was examined by the Friedman test followed by Dunn's multiple comparisons test. To examine the influence of NYT on CBF changes, changes in CBF following administration of each NYT dose were compared with that following 0 g/kg (DW) administration employing the Mann–Whitney test. The calculated *p* value was adjusted with the Bonferroni correction to account for family-wise type I error for multiple comparisons. The influence of the vagus nerve cut and additional ATR injection on CBF change following NYT administration was assessed by the Mann–Whitney test. CBF changes induced by brushing stimulation following DW and NYT administration were compared by the Wilcoxon matched-pairs signed-ranks test. The preadministration baseline was compared with the Mann–Whitney test. Differences with *p* <0.05 were deemed statistically significant. Data are expressed as the median and interquartile range (25%–75%).

## 3. Results

### 3.1. Influence of Intragastric Administration of DW and NYT on Resting CBF (Experiment 1)

**Figure 1:**
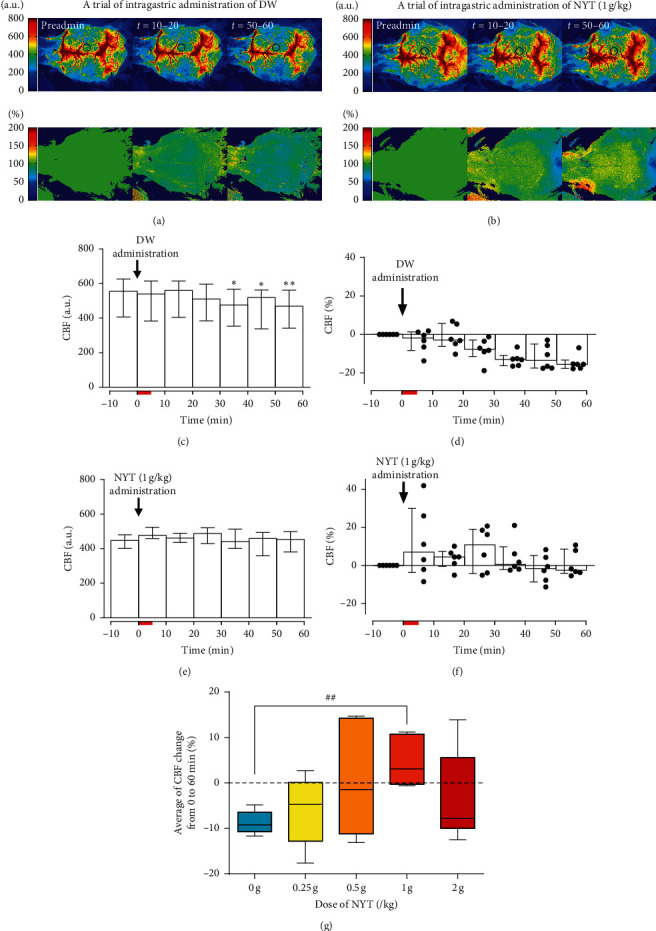
Spatial-temporal changes in cerebral cortex blood flow following intragastric administration of distilled water (DW) (a) and 1 g/kg of ninjin'yoeito (NYT) solution (b). Blood flow images of the dorsal aspect of the mouse cerebral cortex acquired with a laser-speckle contrast imaging system and the obtained images are averaged every 10 min (upper panels in (a) and (b)). The percentage change in cerebral blood flow (CBF) is calculated by dividing each image following intragastric administration by a preadministration image (lower panels in (a) and (b)). Preadmin, preadministration; *t* = 10–20, an averaged image obtained at 10–20 min following the commencement of intragastric administration; *t* = 50–60, an averaged image obtained at 50–60 min following the commencement of administration. Summary of 10 min CBF value is extracted by a region of interest on the right parietal cortex (c), (e). The location of a region of interest (1.5 mm in diameter) is shown as circles in the images in the upper panels of (a) and (b). The CBF change (%) with respect to the preadministration value was calculated (d), (f). The time of administration starting at 0 min is indicated by an arrow in each graph and a thick horizontal line on the time axis. Each dot (●) at each time point in panels (d) and (f) indicates the data of an individual mouse. ^*∗*^*p* < 0.05 and ^*∗∗*^*p* < 0.01 vs. preadministration value, tested by Dunn's test. CBF change (%) between the beginning of administration (0 min) and the end of the measurement (60 min) was averaged and compared (g). In panel (g), a box represents the median and 25%–75% range and whiskers indicate the minimum and the maximum values. ^##^*p* < 0.01 vs. 0 g/kg; evaluated by Mann–Whitney test. The value was adjusted by Bonferroni's method. *n* = 6 in each dose.

Figures 1(a) and 1(b) show examples of CBF images following intragastric administration of DW and NYT (1 g/kg), respectively, on resting CBF. The CBF did not change within 20 min (*t* = 10–20) following the start of DW administration; however, CBF in the cerebral cortex globally decreased (*t* = 50–60; Figure 1(a)). In a trial of NYT (1 g/kg) administration (as shown in Figure 1(b)), such CBF decreases following DW administration were not observed. CBF did not change (*t* = 10–20) or slightly increased in the cerebral cortex (*t* = 50–60; Figure 1(b)). To summarize the 10 min data of CBF in the right parietal cortex (6 mice), the resting CBF did not change within 20–30 min following the start of DW administration (Figure 1(c)) but gradually decreased from 30 to 40 min onward (*p* <0.02). At 50–60 min, CBF was lower than the preadministration level in all mice and reached −15.5% (−17.6% to −13.2%) of preadministration levels (Figure 1(d)). In contrast, CBF did not show consistent changes following NYT (1 g/kg) administration throughout the observation period or the statistically significant difference compared with the preadministration level (Figures 1(e) and 1(f)). In addition to the right parietal cortex, the influence on other regions of the cerebral cortex was tested by examining ROIs on the bilateral frontal, parietal, and occipital cortex, showing that similar CBF changes occurred in either brain region in the right and left hemispheres (Figure S1). Mean arterial pressure was not affected by either solution (Figure S2).

In Experiment 1, doses of 0.25 g/kg, 0.5 g/kg, and 2 g/kg of NYT were also tested (*n* = 6 in each dose). CBF did not significantly change over the recording period for any dose, but we observed that the tendency for CBF to decrease following DW administration tended to attenuate in a dose-dependent manner between the 0.25 g/kg and 1 g/kg doses (Figure S3). For example, CBF at 50–60 min following the start of NYT administration (0.25 g/kg, 0.5 g/kg, and 1 g/kg) was −9.9% (−15.0% to −1.2%), −3.7% (−11.3% to +5.5%), and −2.5% (−4.1% to +8.6%) with respect to preadministration levels, respectively. The maximum dose in the present study (2 g/kg) did not further enhance the effect against CBF decrease (−16.1% (−19.5% to +4.9%)). The average CBF change (%) following administration of each solution dose exhibited a bell-shaped dose-response (Figure 1(g)). The Mann–Whitney test showed that there was a significant difference between DW and 1 g/kg of NYT (−9.2% (−10.7% to −6.4%) vs. +3.1% (−0.3% to +10.8%)), respectively; *p*=0.0088) but not between DW and the other doses of NYT. Preadministration CBF was not different among doses.

### 3.2. Influence of Nerve Transection on the Effect of Intragastric NYT Administration (Experiment 2)

**Figure 2:**
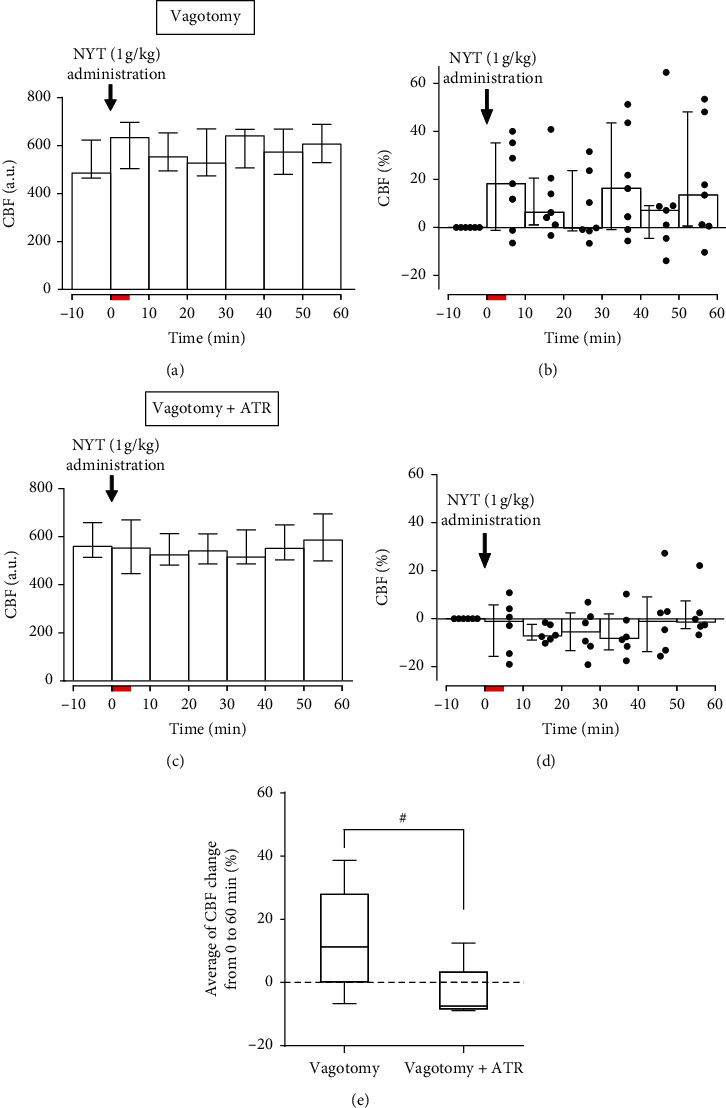
Influence of vagotomy (a), (b) and additional atropine (ATR) (c), (d) on resting cerebral blood flow (CBF) following 1 g/kg of ninjin'yoeito (NYT) administration. ATR was administered after the influence of vagotomy was tested (*n* = 7). One mouse vomited following NYT administration in the vagotomy + ATR condition, and the data were excluded from the analysis. Thus, the number of mice under these conditions was 6. The average CBF change (%) was compared between the vagotomy and vagotomy + ATR conditions (e). ^#^*p* < 0.05; evaluated by Mann–Whitney test. For further details, refer to Figure 1.

Next, to investigate the possibility that neural information on NYT administration in the stomach is involved, we intragastrically administered NYT solution (1 g/kg) to mice vagotomized at the cervical level (*n* = 7). In these mice, CBF increases following NYT administration were observed in a part of the cohort; however, there was no statistically significant change (Figures 2(a) and 2(b)). The average of CBF change in these vagotomized mice was +11.3% (+0.2% to +27.9%) and did not differ from that in the intact vagus nerve mice (Figure 1(g)). Also, the NYT solution was administered to vagotomized and spinalized mice (*n* = 2), showing a slight CBF increase in both mice (Figure S4). CBF changes (%) following NYT solution administration in these mice were +6.4% and +8.4%, which were similar to those changes in the nerve-intact and vagotomized mice.

### 3.3. Influence of mACh Receptor Blocker on the Effect of Intragastric NYT Administration (Experiment 3)

The influence of vagotomy was not obvious in Experiment 2. In these mice, both afferents and efferents of vagus nerves were disconnected; thus, we presumed that the contribution of a vagal cholinergic effect could be excluded. Then, to reduce the number of animals used, we tested the contribution of cerebral mACh receptors to the effect of NYT by systemic administration of mACh receptor blocker on the vagotomized mice used in Experiment 2 (*n* = 7). One of the seven mice vomited during the data collection. Thus, the data following ATR injection of this mouse was excluded from the analysis. In the remaining ATR-treated mice (*n* = 6), there was no statistically significant CBF change following the NYT administration (Figure 2(c)). However, CBF at 10–20 min following NYT administration was lower than the preadministration level in all the mice, and CBF tended to decrease (Figure 2(d)). CBF change (%) following the NYT administration in these mice was −7.5% (−8.4% to +3.3%) and was significantly lower than that in the non-ATR-treated mice (vagotomy only; Figure 2(e)). In contrast, preadministration CBF did not differ between the vagotomized mice and the ATR-treated mice.

### 3.4. Effect of Intragastric NYT Administration on CBF Increase in response to Brushing Stimulation (Experiment 4)

**Figure 3:**
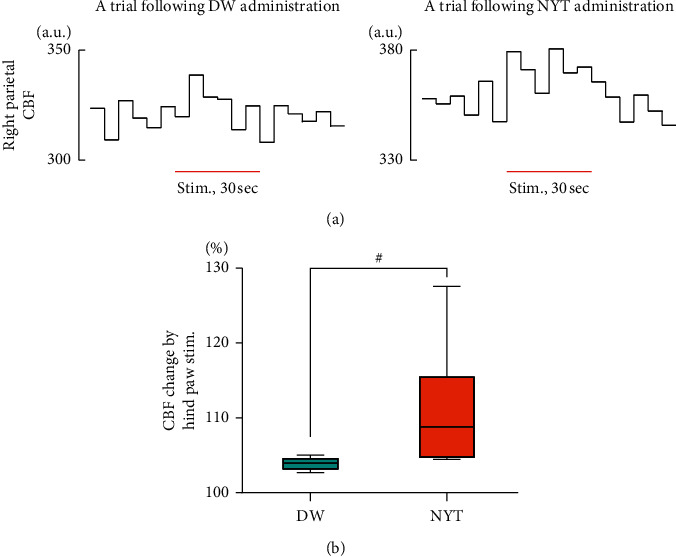
Effect of 1 g/kg of ninjin'yoeito (NYT) administration on cerebral blood flow (CBF) response to brushing stimulation applied to the left hind paw. Time courses of CBF in the right parietal cortex are presented with 5 sec temporal resolution (a). The period of brushing stimulation is indicated by a horizontal line. CBF change (%) was calculated by a maximum CBF value during brushing stimulation with respect to a CBF value immediately before the stimulation, and the CBF changes following distilled water (DW) and NYT administration were compared (b). ^#^*p* < 0.05; tested by Wilcoxon matched-pairs signed-ranks test. a.u.: arbitrary unit and stim.: stimulation. *n* = 6.

In addition to the effect of NYT on resting CBF, the present study examined the effect on CBF increase in response to somatosensory stimulation (*n* = 6). Based on the above-mentioned results, brushing stimulation was applied to the left hind paw at the time when CBF was not affected by intragastric solution administration (at 10–16 min following the start of administration).  Figure 3(a) shows examples of CBF data in the right parietal cortex (contralateral to the stimulation). Following the administration of either DW or NYT (1 g/kg), CBF increased during brushing stimulation and returned to prestimulation levels within 15 sec after the stimulation was terminated. The CBF response following NYT administration was larger than that following DW administration. CBF change (%) during brushing stimulation following NYT administration was significantly larger than that following DW administration (108.8% (104.8% to 115.4%) vs. 104.0% (103.2% to 104.6%), respectively; *p*=0.031; Figure 3(b)).

## 4. Discussion

### 4.1. Homeostatic Effect of NYT on CBF

In the present study, CBF gradually decreased 30 min after the intragastric administration of DW. DW administration did not affect blood pressure (consistent with a previous study [25]), indicating constriction of the cerebral vasculatures. Such a response could be due to stomach distention or osmotic stimuli [25, 26]. Surprisingly, the physiological influence of water drinking on CBF has scarcely been studied, and the mechanism needs to be clarified in the future. The present study found that 1 g/kg of NYT had an antagonistic effect on vasoconstriction. Our result indicated that NYT administration did not affect blood pressure or induce remarkable vasodilation to elevate resting CBF, whereas NYT ameliorated cerebral vasoconstriction by DW administration and had a homeostatic effect of maintaining CBF at a certain level.

Reports on the effect of *in vivo* NYT are mostly studies of multiple administrations. The number of studies with a single administration of NYT is limited; however, it was demonstrated that NYT effects were elicited within 60 min following administration [1]. Therefore, the effects of NYT could be triggered in at least several tens of minutes.

The effect of NYT on resting CBF tended to be dose-dependent, and 1 g/kg of NYT was the most effective in the present study. In contrast, the effect of NYT was rather weaker at a higher dose of 2 g/kg and exhibited a bell-shaped dose-response curve. The result that 1 g/kg of NYT is effective was consistent with a previous study reporting an improvement of cognitive impairment in mice [1]. The following experiments in the present study used the 1 g/kg dose.

### 4.2. Pathway of NYT Effect on CBF

In general, information about chemical stimulation in the gastrointestinal tract is transmitted to the brain via afferents of the vagus nerve [27, 28], whereas the possibility has been suggested that the medicinal properties of NYT act directly on the brain via the bloodstream [1]. Neither vagotomy nor spinalization affected resting CBF following the intragastric administration of NYT in the present study, suggesting that a component of the NYT absorbed from the gastrointestinal tract into the blood affected the CBF regulatory system but not neural information from the gastrointestinal tract. After orally administrating a component (paeoniflorin) of Paeoniae radix, which is a crude drug included in NYT, plasma concentrations of paeoniflorin increased at the latest 5 min after administration and reached a peak at 30 min in rats [29]. Active oxygen-scavenging ability in plasma increased 60 min following 1 g/kg of NYT administered to rats [30]. These results indicate the possibility that a component of administered NYT had been already transferred to the blood during CBF measurement.

### 4.3. Activation Effect of NYT on the Cerebral Cholinergic System

As the result of mACh receptor blocker injection in the vagotomized mice, CBF change following NYT administration decreased. This result indicates that administered NYT humorally reached the brain and exerted a mechanism of CBF control by activating mACh receptors. Somatosensory stimulation increases regional CBF [31–35]. In the present study, brushing stimulation was applied at a time when the resting CBF was not affected following solution administration, and consequently, the CBF increase in the contralateral parietal cortex (i.e., the hind limb area of the primary somatosensory cortex) induced by the stimulation was larger following NYT administration than that following DW administration. This result indicates that NYT enhanced regional CBF increase in response to somatosensory stimulation.

Cholinergic neurons originating in the basal forebrain distribute their axons to the cerebral cortex and hippocampus and increase CBF via muscarinic and nicotinic acetylcholine receptors [7, 8, 10, 11]. The CBF increase induced by brushing stimulation to the hind limb in anesthetized rats was attenuated to two-thirds by inhibiting the nucleus basalis of Meynert (NBM), which projects cholinergic nerve fibers to the cerebral cortex, suggesting the cholinergic system originating from the NBM is partly involved [32, 36]. The result of a previous study [37] showing that CBF increase by somatosensory stimulation is enhanced by NBM stimulation at a subthreshold intensity (i.e., it does not affect CBF) is similar to the present finding that NYT enhanced CBF increase in response to somatic stimulation without a remarkable increase in resting CBF. It has also been reported that a blood flow increase in the olfactory bulb induced by olfactory nerve stimulation is enhanced by pharmacological stimulation of the cholinergic system [24]. The effect of NYT revealed by the present study is not a mere vasodilatory effect; thus, it is probably due to the vasodilatory system, such as the cerebral cholinergic system, rather than NYT having a direct action on the blood vessels.

An *in vitro* study showed that NYT and its constituting crude drug (Polygalactia radix) increase the choline acetyltransferase activity of basal forebrain cells [4], which is important for CBF regulation. Intragastric administration of NYT and Polygalactia radix enhances tremor induced by oxotremorine (a mACh receptor agonist) in mice [1]. Additionally, its effect was abolished by scopolamine hydrobromide, which is blood–brain barrier-permeable, but was not affected by scopolamine methyl bromide, which is not permeable. Therefore, regarding activation of the cerebral cholinergic system by NYT administration, the present result is consistent with the results of the previous studies. In contrast, the detailed mechanism to activate the cerebral cholinergic system by NYT and a potential contribution of other CBF regulatory systems such as nitric oxide/nitric oxide synthase pathway need further investigation.

## 5. Conclusions

The present results suggest that NYT prevents CBF decrease following intragastric administration and enhances CBF increase in response to somatic stimulation. We consider that mACh receptors in the brain possibly contribute to its mechanism, independent from the vagus nerve. Exercise is beneficial for cognitive functions in the elderly. Walking and mastication increase CBF in the neocortex partly by activating the cholinergic system [38, 39]. Therefore, the beneficial effect of physical stimulation on brain circulation and function is expected to be further promoted by combining it with NYT.

## Figures and Tables

**Table 1:** Summary of the experimental protocol.

Experiment #	Recording	Condition	*n*
Experiment 1	Resting CBF	Nerves intact	17^*∗*^
Experiment 2	Resting CBF	Vagotomized	7
		Vagotomized + spinalized	2
Experiment 3	Resting CBF	Vagotomized + ATR	6^*∗∗*^
Experiment 4	Brushing stimulation-evoked CBF change	Nerves intact	6

^*∗*^Arterial blood pressure was recorded in 10 animals. ^*∗∗*^CBF recording was conducted following the recording in vagotomized condition was completed (experiment 2). Data of one mouse was excluded as the mouse vomited during recording.

## Data Availability

The data sets used and/or analyzed during the current study are available from the corresponding author on reasonable request.

## Supplementary Materials

Supplementary Materials Figure S1: cerebral blood flow changes in each cortical region following (a) distilled water and (b) 1 g/kg of ninjin'yoeito solution. CBF values of frontal, parietal, and occipital cortices were extracted by regions of interest (ROIs), and the ROI locations are shown (c): frontal, AP = +2 to +0.5 mm; parietal, AP = 0 to −1.5 mm; occipital, AP = −2 to −3.5 mm from bregma and 0.5 to 2 mm from the midline. The number allocated in each graph refers to the number of ROIs shown in (c). The time of administration starting at 0 min is indicated by an arrow in each graph and a thick horizontal line on the time axis. ^*∗*^*p* < 0.05, ^*∗∗*^*p* < 0.01, ^*∗∗∗*^*p* < 0.001 vs. preadministration value (between −10 and 0 min), tested by Dunn's test; *n* = 6 for each solution. Figure S2: influence of intragastric administration of distilled water and 1 g/kg of ninjin'yoeito solution on mean arterial pressure (MAP). MAP was averaged for over 10 min. The time of administration starting at 0 min is indicated by an arrow in each graph and a thick horizontal line on the time axis. The averaged MAP values are presented in the upper panels, and the %MAP change with respect to the preadministration value is shown in the lower panels; n = 6. Figure S3: dose dependence of ninjin'yoeito (NYT) solution's effect on cerebral blood flow (CBF). A 10-min CBF value was extracted by a region of interest on the right parietal cortex. Administration doses were 0 g/kg (i.e., distilled water; DW) (a), 0.25 g/kg (b), 0.5 g/kg (c), 1 g/kg (d), and 2 g/kg (e). The time of administration starting at 0 min is indicated by an arrow in each graph and a thick horizontal line on the time axis. The obtained CBF values are summarized on the left of each panel. The %CBF change with respect to the preadministration value was calculated and presented in the right of each panel. Each line in the graphs in the right panels indicates data from individual mice. ^*∗*^*p* < 0.05, ^*∗∗*^*p* < 0.01 vs. preadministration value; tested with Dunn's test; n = 6 in each dose. Data of DW and 1 g/kg of NYT are fundamentally the same as those presented in Figure 1(c)–(f), but are re-presented in this figure for comparison purposes Figure S4: influence on resting cerebral blood flow (CBF) following 1 g/kg of ninjin'yoeito (NYT) administration in vagotomized and spinalized mice (*n* = 2). A 10-min CBF value was extracted by a region of interest on the right parietal cortex. The time of administration starting at 0 min is indicated by an arrow in each graph and a thick horizontal line on the time axis. The obtained CBF values are summarized in the left panel and the %CBF change with respect to the preadministration value is presented in the right panel. Each line indicates data from individual mice.
