# Supplementary material for: Influence of Intragastric Administration of Traditional Japanese Medicine, Ninjin'Yoeito, on Cerebral Blood Flow via Muscarinic Acetylcholine Receptors
Source: Evid Based Complement Alternat Med. 2021 Aug 7;2021:9930023. doi: 10.1155/2021/9930023 (PMC8367494; doi:10.1155/2021/9930023)
Supplement: Supplementary Materials — Figure S1: cerebral blood flow changes in each cortical region following (a) distilled water and (b) 1 g/kg of ninjin'yoeito solution. CBF values of frontal, parietal, and occipital cortices were extracted by regions of interest (ROIs), and the ROI locations are shown (c): frontal, AP = +2 to +0.5 mm; parietal, AP = 0 to −1.5 mm; occipital, AP = −2 to −3.5 mm from bregma and 0.5 to 2 mm from the midline. The number allocated in each graph refers to the number of ROIs shown in (c). The time of administration starting at 0 min is indicated by an arrow in each graph and a thick horizontal line on the time axis. ∗p < 0.05, ∗∗p < 0.01, ∗∗∗p < 0.001 vs. preadministration value (between −10 and 0 min), tested by Dunn's test; n = 6 for each solution. Figure S2: influence of intragastric administration of distilled water and 1 g/kg of ninjin'yoeito solution on mean arterial pressure (MAP). MAP was averaged for over 10 min. The time of administration starting at 0 min is indicated by an arrow in each graph and a thick horizontal line on the time axis. The averaged MAP values are presented in the upper panels, and the %MAP change with respect to the preadministration value is shown in the lower panels; n = 6. Figure S3: dose dependence of ninjin'yoeito (NYT) solution's effect on cerebral blood flow (CBF). A 10-min CBF value was extracted by a region of interest on the right parietal cortex. Administration doses were 0 g/kg (i.e., distilled water; DW) (a), 0.25 g/kg (b), 0.5 g/kg (c), 1 g/kg (d), and 2 g/kg (e). The time of administration starting at 0 min is indicated by an arrow in each graph and a thick horizontal line on the time axis. The obtained CBF values are summarized on the left of each panel. The %CBF change with respect to the preadministration value was calculated and presented in the right of each panel. Each line in the graphs in the right panels indicates data from individual mice. ∗p < 0.05, ∗∗p < 0.01 vs. preadministration value; tested with Dunn's test; n = [file 9930023.f1.docx]

## Supplementary Materials


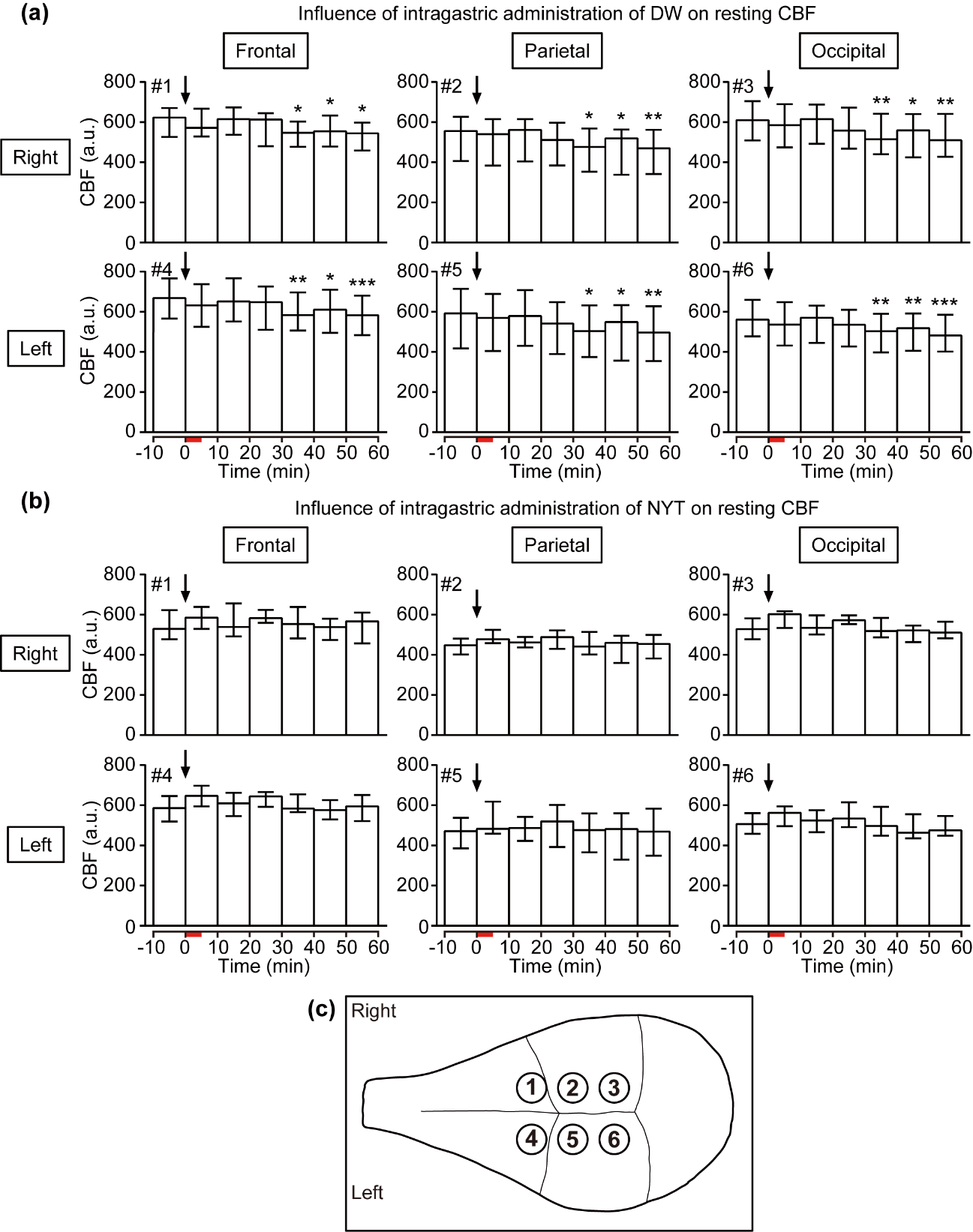


Figure S1: Cerebral blood flow (CBF) changes in each cortical region following distilled water (DW) (a) and 1 g/kg of ninjin’yoeito (NYT) solution (b). CBF values of frontal, parietal, and occipital cortices were extracted by regions of interest (ROIs), and the ROI locations are shown (c): frontal, AP = +2 to +0.5 mm; parietal, AP = 0 to −1.5 mm; occipital, AP = −2 to −3.5 mm from Bregma and 0.5 to 2 mm from the midline. The number allocated in each graph refers to the number of ROIs shown in (c). The time of administration starting at 0 min is indicated by an arrow in each graph and a thick horizontal line on the time axis. * p < 0.05, ** p < 0.01, *** p < 0.001 vs. preadministration value (between −10 and 0 min); tested by Dunn’s test. n = 6 for each solution.


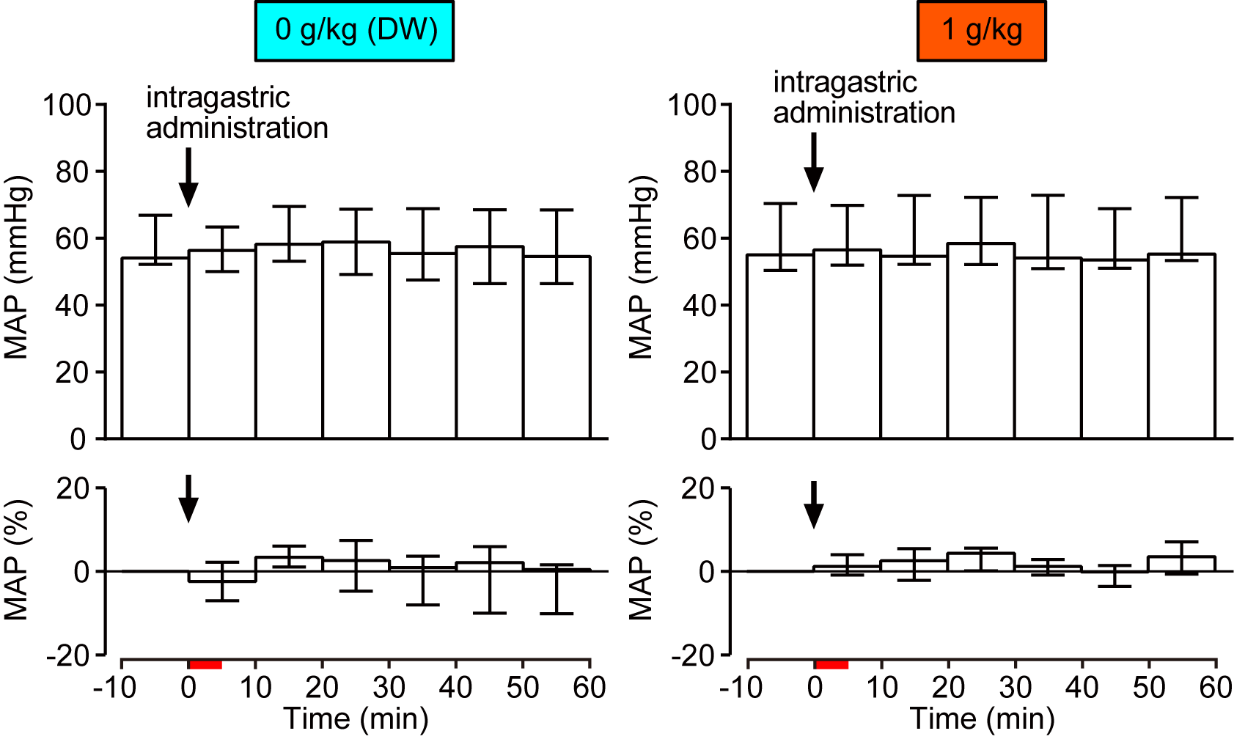


Figure S2: Influence of intragastric administration of distilled water (DW) and 1 g/kg of ninjin’yoeito (NYT) solution on mean arterial pressure (MAP). MAP was averaged for over 10 min. The time of administration starting at 0 min is indicated by an arrow in each graph and a thick horizontal line on the time axis. The averaged MAP values are presented in the upper panels, and the % MAP change with respect to the preadministration value is shown in the lower panels. n = 6.


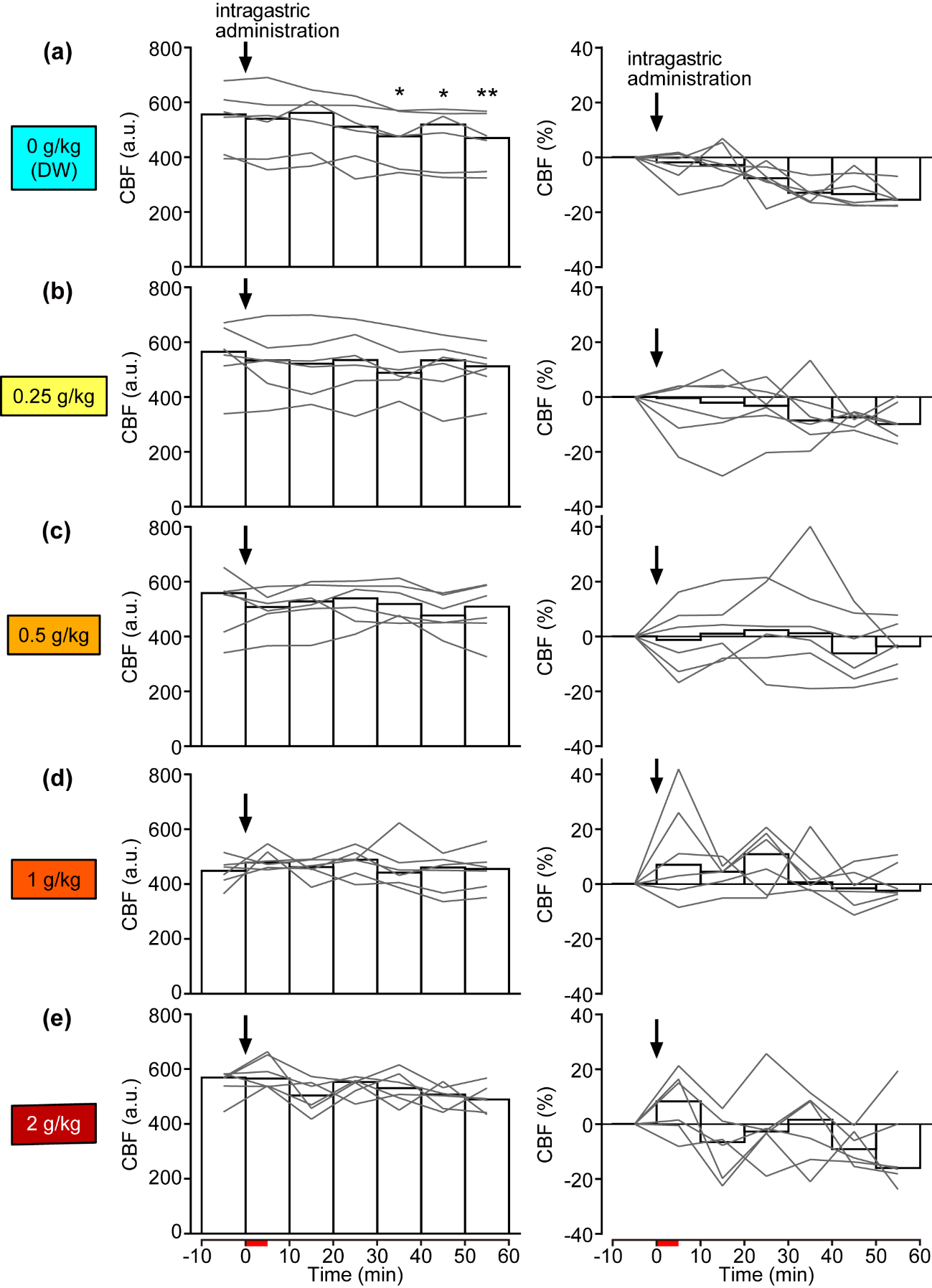


Figure S3: Dose dependence of ninjin’yoeito (NYT) solution’s effect on cerebral blood flow (CBF). A 10-min CBF value was extracted by a region of interest on the right parietal cortex. Administration doses were 0 g/kg (i.e., distilled water; DW) (a), 0.25 g/kg (b), 0.5 g/kg (c), 1 g/kg (d), and 2 g/kg (e). The time of administration starting at 0 min is indicated by an arrow in each graph and a thick horizontal line on the time axis. The obtained CBF values are summarized on the left of each panel. The % CBF change with respect to the preadministration value was calculated and presented in the right of each panel. Each line in the graphs in the right panels indicates data from individual mice. * p < 0.05, ** p < 0.01 vs. preadministration value; tested with Dunn’s test. n = 6 in each dose. Data of DW and 1 g/kg of NYT are fundamentally the same as presented in Figure 1 (c)-(f), but are re-presented in this figure for comparison purposes.


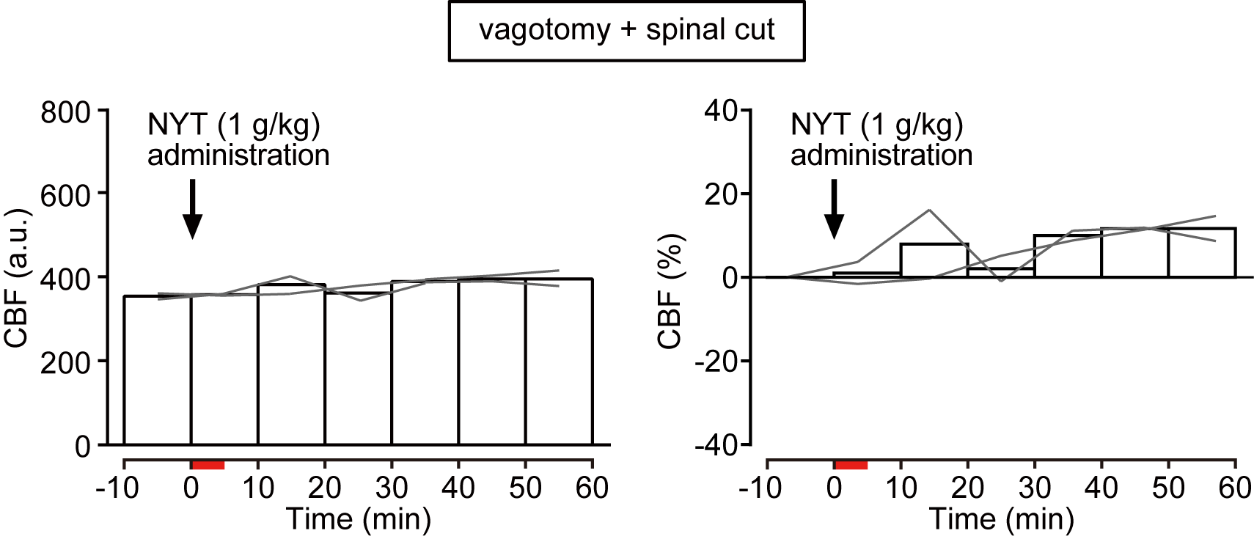


Figure S4. Influence on resting cerebral blood flow (CBF) following 1 g/kg of ninjin’yoeito (NYT) administration in vagotomized and spinalized mice (n = 2). A 10-min CBF value was extracted by a region of interest on the right parietal cortex. The time of administration starting at 0 min is indicated by an arrow in each graph and a thick horizontal line on the time axis. The obtained CBF values are summarized in the left panel and the % CBF change with respect to the preadministration value is presented in the right panel. Each line indicates data from individual mice.
